# Malignant T Cell Activation by a *Bacillus* Species Isolated from Cutaneous T-Cell Lymphoma Lesions

**DOI:** 10.1016/j.xjidi.2021.100084

**Published:** 2021-12-16

**Authors:** Carina A. Dehner, William E. Ruff, Teri Greiling, Márcia S. Pereira, Sylvio Redanz, Jennifer McNiff, Michael Girardi, Martin A. Kriegel

**Affiliations:** 1 Department of Immunobiology, Yale University School of Medicine, New Haven, Connecticut, USA; 2 Department of Pathology & Immunology, Washington University School of Medicine in St. Louis, St. Louis, Missouri, USA; 3 Department of Dermatology, Oregon Health & Science University, Portland, Oregon, USA; 4 Department of Translational Rheumatology and Immunology, Institute of Musculoskeletal Medicine, University of Münster, Münster, Germany; 5 Department of Dermatopathology, Yale University School of Medicine, New Haven, Connecticut, USA; 6 Department of Dermatology, Yale University School of Medicine, New Haven, Connecticut, USA; 7 Section of Rheumatology and Clinical Immunology, Department of Medicine, University Hospital Münster, Münster, Germany

**Keywords:** ASV, amplicon sequence variant, CLA, cutaneous lymphocyte‒associated antigen, CTCL, cutaneous T-cell lymphoma, MF, mycosis fungoides, rRNA, ribosomal RNA, STAT3, signal transducer and activator of transcription 3

## Abstract

Cutaneous T-cell lymphoma (CTCL) is a life-debilitating malignancy of lymphocytes homing to the skin. Although CTCL is thought to arise from a combination of genetic, epigenetic, and environmental factors, specific triggers are unclear. The skin is colonized by a unique microbiota and is heavily influenced by its interactions. We hypothesized that adaptive immune responses to skin commensals lead to clonal T-cell proliferation and transformation in the appropriate genetic background. We therefore collected lesional and nonlesional skin microbiota from patients with CTCL to study T cell interactions using skin T cell explants and peripheral, skin-homing CD4^+^ T cells. By various methods, we identified *Bacillus safensis* in CTCL lesions, a rare human commensal in healthy skin, and showed that it can induce malignant T cell activation and cytokine secretion. Taken together, our data suggest microbial triggers in the skin microbiota of patients with CTCL as potential instigators of tumorigenesis.

## Introduction

Cutaneous T-cell lymphoma (CTCL) is a cancer of skin-homing T cells, with mycosis fungoides (MF) being the most common subtype. CTCL is conceptually similar to mucosa-homing lymphocytes in mucosa-associated lymphoid tissue lymphoma (Girardi et al., 2004; Isaacson and Du, 2004). Malignant T cells are clonal and proliferate in discrete histological clusters known as Pautrier’s microabscesses within the epidermis (Robson, 2007), presenting as scaly skin patches and plaques (Girardi et al., 2004). With MF often presenting as a fairly indolent, however life-debilitating disease, diagnosis is challenging and requires tissue sampling, which may not routinely be performed in the early stages of the disease.

Despite attempts to understand disease heterogeneity, the pathogenesis remains poorly understood. Previous studies associated CTCL with HLA class II alleles (Jackow et al., 1996), supporting the already postulated hypothesis of putative antigenic triggers being needed for T cell activation and transformation (Tan et al., 1974). Interestingly, a recent study showed that an intensive regimen of intravenously given antibiotics followed by oral application ameliorated CTCL disease activity (Lindahl et al., 2019), suggesting that microbial triggers are involved in tumorigenesis. Furthermore, an animal model of CTCL supports the involvement of the microbiome because germ-free conditions resulted in significantly milder disease, which was reversed by cohousing with specific pathogen-free housed mice (Fanok et al., 2018). Despite these studies, the contribution of skin commensal antigens to human disease remains unknown. We hypothesize that an antigenic signal from the skin microbiome triggers the activation and transformation of skin-homing T cells similar to that seen in *Helicobacter pylori‒*promoted mucosa-associated lymphoid tissue lymphoma (Girardi et al., 2004; Isaacson and Du, 2004).

To investigate the role of the skin microbiome in CTCL, we performed exploratory 16S ribosomal RNA (rRNA) V1‒V3 sequencing on lesional and nonlesional skin microbiomes from seven patients with CTCL and anatomically corresponding sites in five healthy subjects (Table 1). In addition, lesional and nonlesional patient-isolated bacteria were cultured from five CTCL skin swabs. T cell isolates from lesional CTCL skin biopsies showed increased signal transducer and activator of transcription 3 (STAT3) phosphorylation compared with nonlesional T cells, which is a characteristic of malignant CTCL T cell populations (Netchiporouk et al., 2014; Sommer et al., 2004). Utilizing these lesional T-cell isolates as well as skin-homing peripheral T cells from patients with CTCL, we identified *Bacillus safensis* but not other patient-isolated skin bacteria as a potential source of antigenic stimuli driving the clonal proliferation of malignant CTCL T cells. Furthermore, cytokine analysis of the cutaneous T cells proliferating in response to *B. safensis* revealed an active inflammatory response, which was not present in nonproliferative cells. These data highlight that skin commensal bacteria may contribute to CTCL pathogenesis and may synergize with other toxigenic bacteria, such as *Staphylococcus aureus* (Blümel et al., 2019; Willerslev-Olsen et al., 2016, 2013), to drive the antigenic and nonantigenic stimulation of malignant T cells that emerge in early CTCL.

**Table 1:** CTCL Patient Cohort

Subject ID	Age	Sex	Diagnosis	Ethnicity	Subtype	Therapy	Phototherapy	Status at Sampling Date	TCR Rearrangement by PCR	Positive for *Bacillus safensis*
MF01	38	F	MF	White	Follicular-type CTCL	–	+	Visible lesions (arm)	TCR skin (+) TCR blood (‒)	By ASV (lesional)
MF02	73	F	MF	White	CTCL stage IB	–	+	Visible lesions (foot)	TCR skin (+) TCR blood (‒)	–
MF03	70	M	MF	White	Follicular-type CTCL	Bexarotene Mechloreth-amine	‒	Visible lesions (leg)	TCR skin (+) TCR blood (‒)	By ASV (lesional)
MF04	62	F	MF	White	CTCL stage IB	Bexarotene Mechloreth-amine	+	Visible lesions (arm)	TCR skin (+) TCR blood (+)	By ASV (lesional and nonlesional)
MF05	77	M	MF	White	CTCL stage IB	–	+	Visible lesions (leg, foot)	TCR skin (NA) TCR blood (‒)	By ASV, culture and FISH
MF06	65	M	MF	White	Follicular-type CTCL	Bexarotene Mechloreth-amine	+	Visible lesions (arm)	TCR skin (NA) TCR blood (‒)	By FISH (lesional)
MF07	62	F	MF	White	CTCL	–	+	Visible lesions (arm)	TCR skin (+) TCR blood (‒)	By ASV (nonlesional) and FISH (lesional)
HD01	32	F	Healthy	White	Negative	No	No	NA	NA	No
HD02	60	F	Healthy	White	Negative	No	No	NA	NA	No
HD03	48	F	Healthy	White	Negative	No	No	NA	NA	No
HD04	42	M	Healthy	Indian	Negative	No	No	NA	NA	No
HD05	45	F	Healthy	White	Negative	No	No	NA	NA	No

Abbreviations: ASV, amplicon sequence variant; CTCL, cutaneous T-cell lymphoma; F, female; HD, healthy donor; ID, identification; M, male; MF, mycosis fungoides; NA, not assessed.

Characteristics of patient and control cohort composed of subject ID, age, sex, diagnosis, ethnicity, subtype, therapy, phototherapy, status at sampling date, and TCR rearrangement by PCR. Mechlorethamine is a topical alkylating drug, and bexarotene is a topical retinoid.

## Results

### 16S rRNA sequence- and culture-based analyses of the CTCL skin microbiota

**Figure 1:**
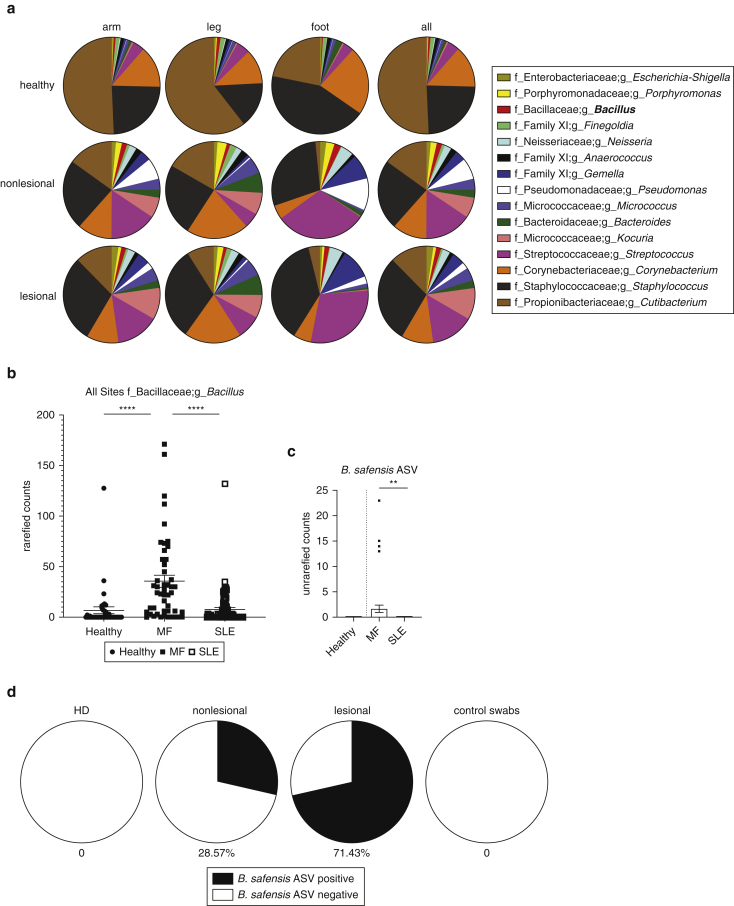
**Characterization of skin microbiome of patients with CTCL using 16S rRNA sequencing.** (**a**) Pie charts depicting the genera that are at least 1% of total genera in the dataset. A total of 15 genera met this criterion and are shown, representing 78.68% of overall 16S rRNA V1‒V3 reads at the genus level. Genera are shown as averages. Skin swabs from the arms of healthy subjects, n = 4; lesional/nonlesional skin swabs of the arms of subjects with CTCL, n = 4, swabs, n = 5. Skin swabs from the legs of healthy subjects, n = 5, swabs, n = 5; lesional/nonlesional skin swabs of the legs of subjects with CTCL, n = 6, swabs, n = 12. Swabs from the feet of healthy subjects, n = 4, swabs, n = 4; Lesional/nonlesional skin swabs of the feet of subjects with CTCL, n = 2, swabs, n = 5. All nonlesional and lesional samples represent paired CTCL samples. f indicates family, and g indicates genus. (**b**) Total number of *Bacillus* genera ASV counts in MF samples (n = 48) compared with those in HD skin swabs (n = 40) and SLE skin swabs (n = 76); *P* = 7.504e–008 healthy versus MF; *P* = 9.629e–009 MF versus SLE. (**c**) *B. safensis* ASV unrarefied counts from healthy (n = 40), MF (n = 48), and SLE skin swabs (n = 76); *P* = 0.0605 healthy versus MF; *P* = 0.0076 MF versus SLE. (**d**) Pie charts representing the percentage of subjects positive for *B. safensis* ASVs, defined as having a 16S rRNA V1‒V3 sequence homology (>99%) to CTCL. Nonlesion/lesion indicate CTCL skin swabs from nonlesional or lesional sites; control swabs indicate air swabs taken before sampling skin sites. ASV, amplicon sequence variant; CTCL, cutaneous T-cell lymphoma; HD, healthy donor; MF, mycosis fungoides; rRNA, ribosomal RNA; SLE, systemic lupus erythematosus. *P*-values were calculated using the unpaired two-tailed Student’s *t*-test. Significance levels are indicated by asterisks: ∗*P* < 0.05; ∗∗*P* < 0.01; ∗∗∗*P* < 0.001; ∗∗∗∗*P* < 0.0001.

**Figure 2:**
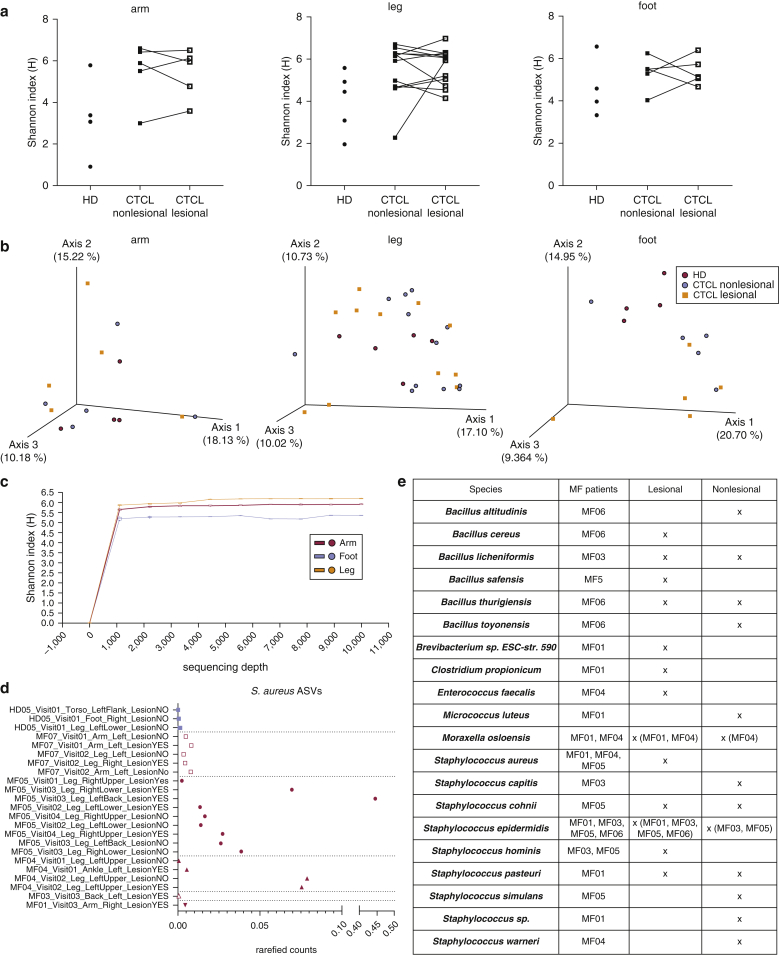
**Alpha and beta diversity of CTCL and HD skin microbiomes as well as culture isolates from lesional and nonlesional CTCL skin.** (**a**) Alpha diversity as measured by Shannon‒Weiner diversity index between skin swabs of arms of healthy subjects, n = 4, swab n = 4; lesional/nonlesional skin swabs of arms from subjects with CTCL, n = 4, swabs, n = 5. Skin swabs of legs from healthy subjects, n = 5, swabs, n = 5; lesional/nonlesional skin swabs of legs from subjects with CTCL, n = 6, swabs, n = 12. Skin swabs of feet from healthy subjects, n = 4, swabs, n = 4; lesional/nonlesional skin swabs of feet from subjects with CTCL, n = 2, swabs, n = 5. All nonlesional and lesional samples represent paired CTCL samples. No statistical difference in Shannon diversity was found. (**b**) Beta diversity as measured by principal-coordinate analysis of unweighted UniFrac distances. The blue sphere indicates healthy, the red sphere indicates nonlesional CTCL, and the orange square indicates lesional CTCL. No statistical difference was found between CTCL and HD unweighted UniFrac with the exception of the foot (PERMANOVA 999 permutations *P* = 0.034). (**c**) Alpha rarefaction curve showing all samples from the arm, leg, and foot to a sequencing depth of 10,000 reads. (**d**) Relative abundance of rarefied counts of *Staphylococcus aureus* ASV in healthy (n = 3) and MF (n = 20) skin swabs color coded by subject. (**e**) Single colony culturing from lesional and nonlesional swabs of patients with CTCL. Lesional and nonlesional sites were sterilely swabbed and then cultured in two different growth media as described in Materials and Methods. Approximately 1 of every 100 colonies per plate or quarter plate was picked and then sequenced. The table shows an overview of bacterial strains per individual, all strains with at least one single colony per swab. All results represent the colonies identified under aerobic growth. Anaerobic media did not show any growth in patient samples or controls. Patient MF07 did not provide samples for culturing. ASV, amplicon sequence variant; CTCL, cutaneous T-cell lymphoma; HD, healthy donor; MF, mycosis fungoides.

**Figure 3:**
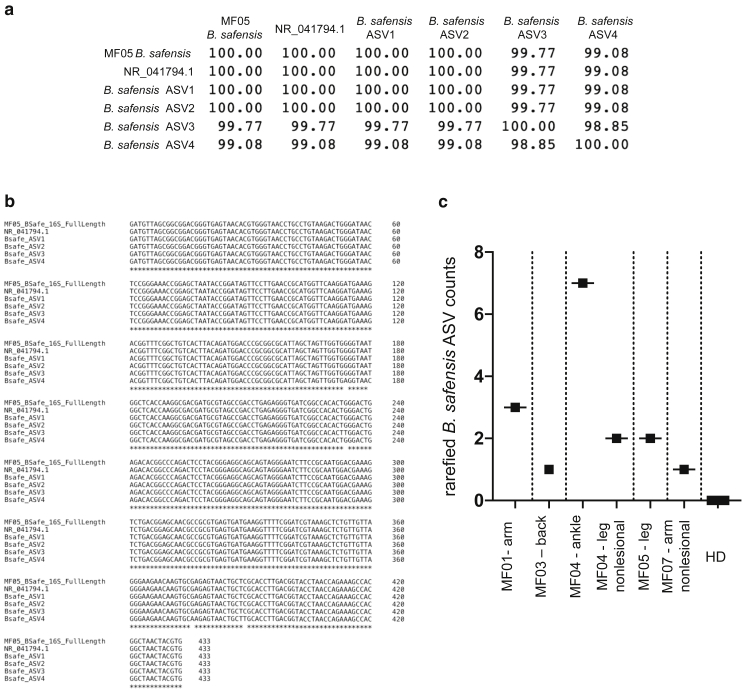
**Alignments and rarefied counts of ASVs from *Bacillus safensis.*** (**a, b**) The 16S rRNA V1‒V3 ASVs were aligned using Clustal omega to corresponding 16S rRNA V1‒V3 of a *B. safensis* culture isolate from a patient with CTCL (MF05) and a representative *B. safensis* strain (FO-36b 16S rRNA NR_041794.1). (**a**) Percent identity matrix showing aligned sequence percent identity relative to each other. (**b**) Aligned nucleotide sequences in the same order as in **a**. *B. safensis* strain FO-36b is listed in this figure as NR_041794.1. (**c**) Number of ASV counts from **a** present in patients with CTCL (four lesional and two nonlesional hits as indicated) compared with that of absent counts in healthy donors (n = 5). ASV, amplicon sequence variant; CTCL, cutaneous T-cell lymphoma; HD, healthy donor; MF, mycosis fungoides; rRNA, ribosomal RNA.

**Figure 4:**
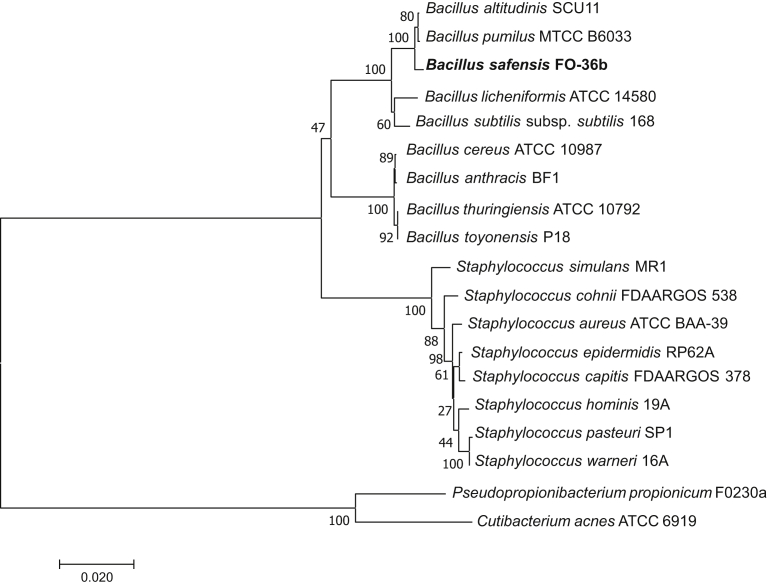
**Evolutionary relationships of *Bacillus* species.** The phylogenetic relationship of the *Bacillus* species identified in this study and related types of strains as well as the identified staphylococci and other skin commensals was examined. The evolutionary history was inferred using the Minimum Evolution method (Rzhetsky and Nei, 1992). The optimal tree with the sum of branch length = 0.35505038 is shown. The percentage of replicate trees in which the associated taxa clustered together in the bootstrap test (100 replicates) are shown next to the branches (Felsenstein, 1985). The tree is drawn to scale, with branch lengths in the same units as those of the evolutionary distances used to infer the phylogenetic tree. The evolutionary distances were computed using the Maximum Composite Likelihood method (Tamura et al., 2004) and are in the units of the number of base substitutions per site.

Rarefied 16S rRNA V1‒V3 analysis of the three most commonly affected body sites (arm, leg, and foot) indicated gross differences at the genus level between the subjects and body sites (Figures 1a and 2 and Table 1), but commonly used metrics of alpha and beta diversity analysis did not indicate significant differences in microbial skin communities between CTCL lesions, nonlesions, and healthy donors (Figure 2a and b). Skin swab analyses revealed the expected prevalence of *Staphylococcus* species (Figure 1a), including *S. aureus* (Figure 2d and e) (Oh et al., 2012), which may contain Staphylococcal enterotoxin involved in CTCL pathogenesis (Willerslev-Olsen et al., 2016). Unexpectedly, combining sequence-based analyses with a culture-based approach revealed low but unique outgrowth of *Bacillus* strains in MF skin (Figures 1b‒d and 2e). The 16S rRNA analysis from two independent cohorts of healthy subjects and subjects with systemic lupus erythematosus studied previously (Greiling et al., 2018; Ruff et al., 2019) indicated a highly significant increase in the genus *Bacillus* in CTCL compared with that in healthy controls and systemic lupus erythematosus (Figure 1b and c). Skin lesion cultures support that this increase is likely due to the presence of multiple *Bacillus* species (Figure 2e). On further analysis of these *Bacillus* species, we identified exclusive 16S rRNA V1‒V3 amplicon sequence variants (ASVs) in five of seven patients with CTCL with >99% identity to a lesional isolate of *Bacillus safensis* and the representative *B. safensis* strain FO-36b (Figure 3a‒c). A phylogenetic tree analysis showed the close relationship of the identified *Bacillus* species (Figure 4), including reference species that are not related to those used in this study.

Consistent with a relatively unique enrichment of *B. safensis* in CTCL, the *Bacillus* genus is generally at low abundance in healthy human skin microbiomes (0.05% of 387 skin samples publicly accessible in the 16S rRNA V1‒V3 Human Microbiome Project database compared with 1.14% in 48 MF skin samples from our cohort). However, genus-level comparisons do not capture the highly selective detection of *B. safensis* in MF skin (predominantly lesional over nonlesional) compared with body site‒matched skin samples from healthy controls (Figure 1c and d). Of note, *B. safensis* was also not identified in a large metagenomic dataset of healthy skin (Oh et al., 2014), suggesting potentially a unique role for this commensal in early CTCL.

### Cutaneous T cells proliferate in response to bacterial antigen

**Figure 5:**
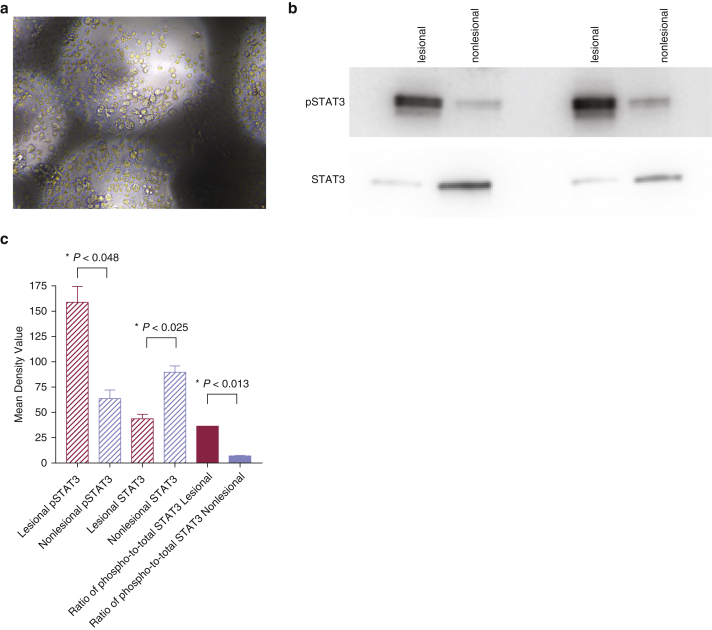
**Isolation of CTCL T cells from skin biopsies and immunoblotting for phosphorylated STAT3.** (**a**) A representative image from a Petri culture dish showing malignant T cells isolated from patient skin biopsy that are growing on collagen-coated grids. (**b, c**) Western blotting shows higher phosphorylation of STAT3 in cutaneous lesional T cells than in cutaneous nonlesional T cells after loading equal amounts of total protein per lane. The blot is representative of two replicates. CTCL, cutaneous T-cell lymphoma; phospho, phosphorylated; pSTAT3, phosphorylated signal transducer and activator of transcription 3; STAT3, signal transducer and activator of transcription. *P*-values were calculated using the unpaired two-tailed Student’s *t*-test. Significance levels are indicated by asterisks: ∗*P* < 0.05.

Because transformation and proliferation of clonal T-cell populations are known to initiate CTCL (Girardi et al., 2004), we next assessed whether patient-isolated bacteria would trigger T-cell activation of malignant T cells extracted from skin lesions. To this end, T cells from two biopsies from patients with CTCL were isolated, harvested, and seeded for T-cell stimulation assays as previously described (Geiger et al., 2009; Greiling et al., 2018; Ruff et al., 2019) (Figure 5a). Cultured T cells were confirmed to express phosphorylated STAT3 (Tyr705) by western blot (Figure 5b), consistent with previous reports showing STAT3 tyrosine phosphorylation within the malignant CTCL T-cell population (Netchiporouk et al., 2014; Sommer et al., 2004). Moreover, STAT3 phosphorylation was increased in lesional compared with that in nonlesional skin, further supporting that malignant T cells were used for subsequent in vitro studies (Figure 5b and c) (Girardi et al., 2004).

**Figure 6:**
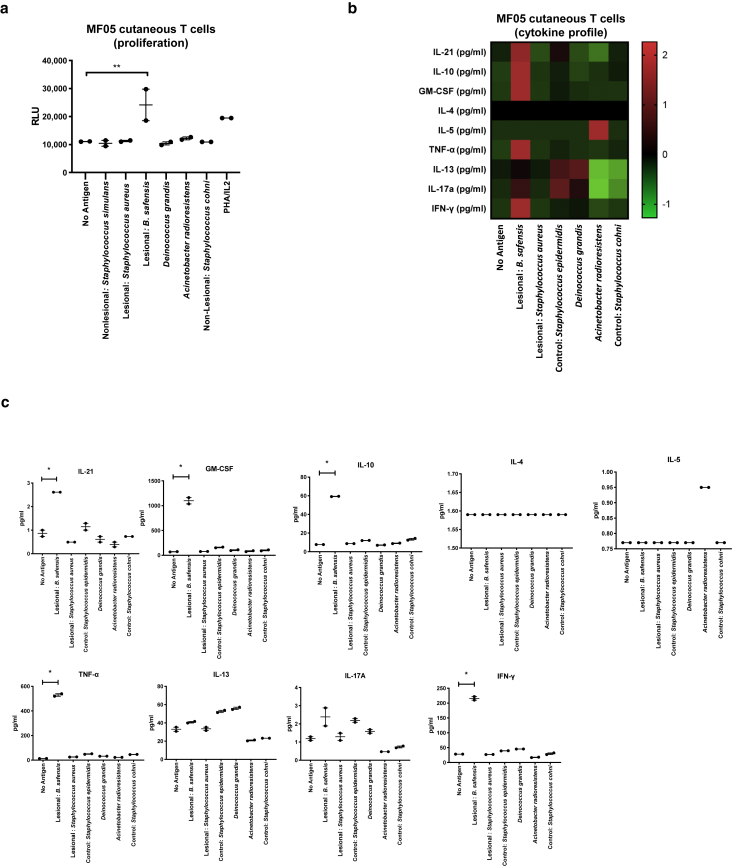
**In vitro T cell proliferation and cytokine studies using patient-isolated cutaneous T cells.** (a) Proliferative responses of human malignant T cells isolated from skin biopsies (MF05) to bacteria isolated from lesional compared with that from nonlesional (control) regions (2 inches next to each matched lesion). Y-axis indicates proliferation as RLUs using a nonradioactive ATP release assay. (**b, c**) Cytokine concentrations (in pg/ml) from the supernatant of the cutaneous T cells stimulated for 72 hours with bacteria as indicated, represented as (**b**) Z-score and (**c**) individual graphs. Functional phenotypes were characterized by cytokine secretion of IL-21, GM-CSF, IFN-γ, TNF-α, IL-17A, IL-4, IL-5, IL-13, and IL-10, respectively. Data points represent duplicates. *P*-values were calculated using the unpaired two-tailed Student’s *t*-test. Significance levels are indicated by asterisks: ∗*P* < 0.05; ∗∗*P* < 0.01. ATP, adenosine triphosphate; MF, mycosis fungoides; RLU, reactive light unit.

**Figure 7:**
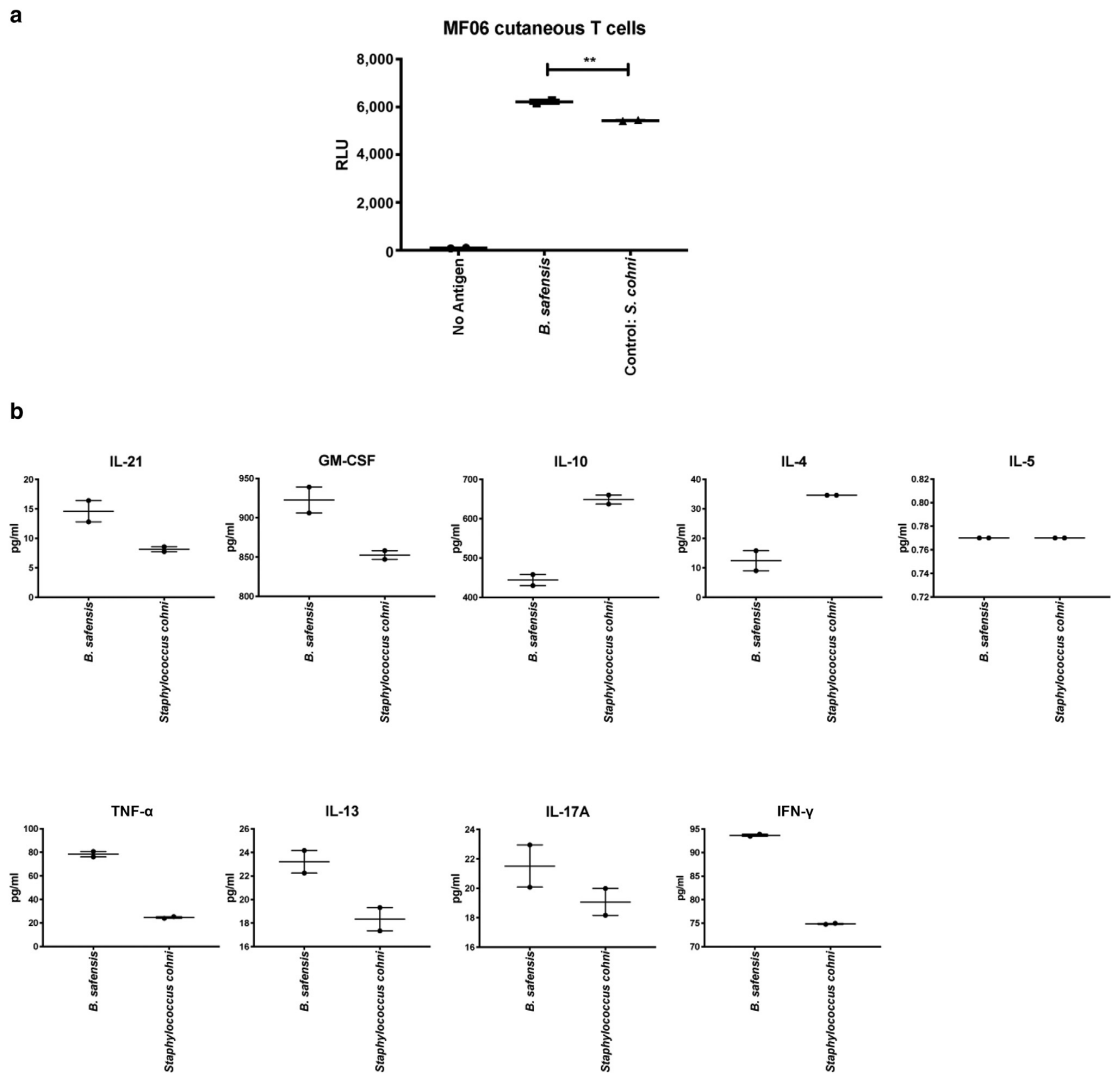
**In vitro T cell cytokine studies using patient-isolated cutaneous T cells.** (**a**) Proliferative responses of human malignant T cells isolated from skin biopsies (MF06) to bacteria isolated from lesional compared with that from nonlesional (control) regions (2 inches next to each matched lesion). Y-axis indicates proliferation as RLUs using a nonradioactive ATP release assay. (**b**) Individual cytokine graphs with cytokine concentrations (in pg/ml) from the supernatant of the cutaneous T cells stimulated for 72 hours with heat-killed bacteria as indicated. Functional phenotypes were characterized by cytokine secretion of IL-21, GM-CSF, IFN-γ, TNF-α, IL-17A, IL-4, IL-5, IL-13, and IL-10, respectively. Data points represent duplicates. *P*-values were calculated using the unpaired two-tailed Student’s *t*-test. Significance levels are indicated by asterisks: ∗*P* < 0.05; ∗∗*P* < 0.01. ATP, adenosine triphosphate; MF, mycosis fungoides; RLU, reactive light unit.

**Figure 8:**
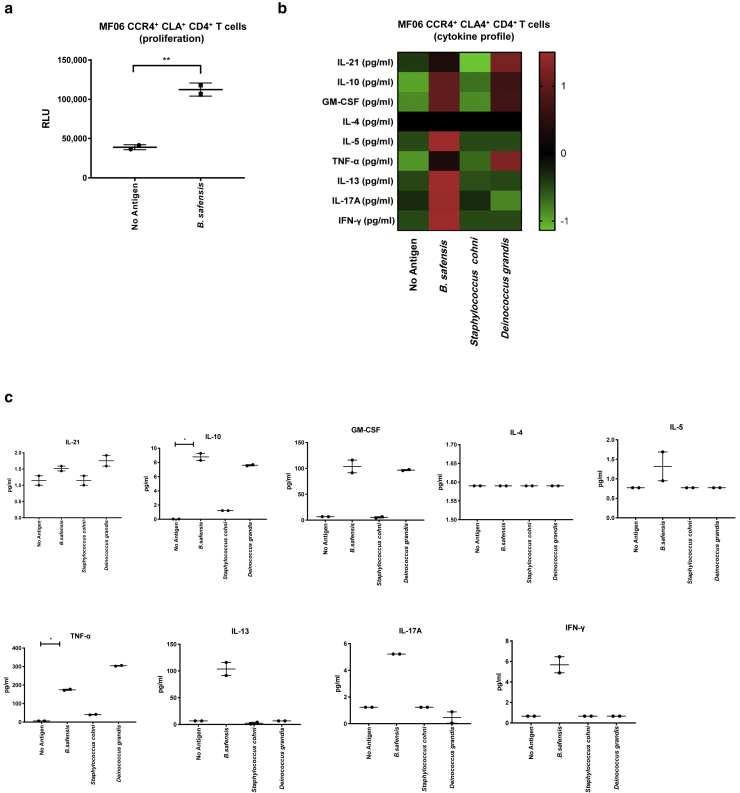
**In vitro T cell proliferation and cytokine studies using patient-isolated peripheral blood T cells.** (**a**) Human CD4^+^ T cells isolated from patient peripheral blood (MF06) selected for skin-homing markers CCR4 and CLA show proliferation to *Bacillus safensis* compared with unstimulated T cells. Y-axis indicates proliferation as RLUs using a nonradioactive ATP release assay. (**b, c**) Cytokine concentrations (in pg/ml) from the supernatant of the cutaneous T cells stimulated for 72 hours with bacteria as indicated, represented as (**b**) Z-score and (**c**) individual graphs. Functional phenotypes were characterized by cytokine secretion of IL-21, GM-CSF, IFN-γ, TNF-α, IL-17A, IL-4, IL-5, IL-13, and IL-10, respectively. Data points represent duplicates. *P*-values were calculated using the unpaired two-tailed Student’s *t*-test. Significance levels are indicated by asterisks: ∗*P* < 0.05; ∗∗*P* < 0.01. ATP, adenosine triphosphate; CLA, cutaneous lymphocyte‒associated antigen; MF, mycosis fungoides; RLU, reactive light unit.

Cutaneous T cells showed a proliferative response in vitro to *B. safensis* (Figure 6a and 7a) but not to other patient-isolated skin bacteria and unrelated bacteria (*Deinococcus grandis* and *Acinetobacter radioresistens*) or to unpulsed monocytes that served as negative controls (Figure 6a). Furthermore, we screened peripheral blood T cells from patient MF06 who was positive for *B. safensis* ASVs in skin swabs and other phylogenetically related *Bacillus* strains by skin culture. Skin-homing, cutaneous lymphocyte‒associated antigen (CLA)^+^, CCR4^+^ CD4^+^ (Clark et al., 2006a; Ferran et al., 2013) T cells proliferated in response to *B. safensis* (Figure 8a), which suggests homing of *Bacillus* antigen‒reactive T cells to CTCL lesions in this individual. To determine the functional polarization of these commensal-reactive T cells, cytokine secretion was measured by a multiplex bead assay as previously published (Greiling et al., 2018; Ruff et al., 2019) (Figures 6b and c, 7b and 8b and c). Most *B. safensis*‒reactive T cells displayed high levels of IL-17A, IL-21, GM-CSF, IFN-γ, IL-10, and TNF-α. These data support an active inflammatory response after bacterial stimulation with *B. safensis*. This phenotype was prevalent in the cutaneous T cells as well as in peripheral skin‒homing (CLA^+^) T cells (Figures 6b and c, 7b and 8b and c) in all patients examined. Significance levels are indicated by asterics: ∗*P* < 0.05.

**Figure 9:**
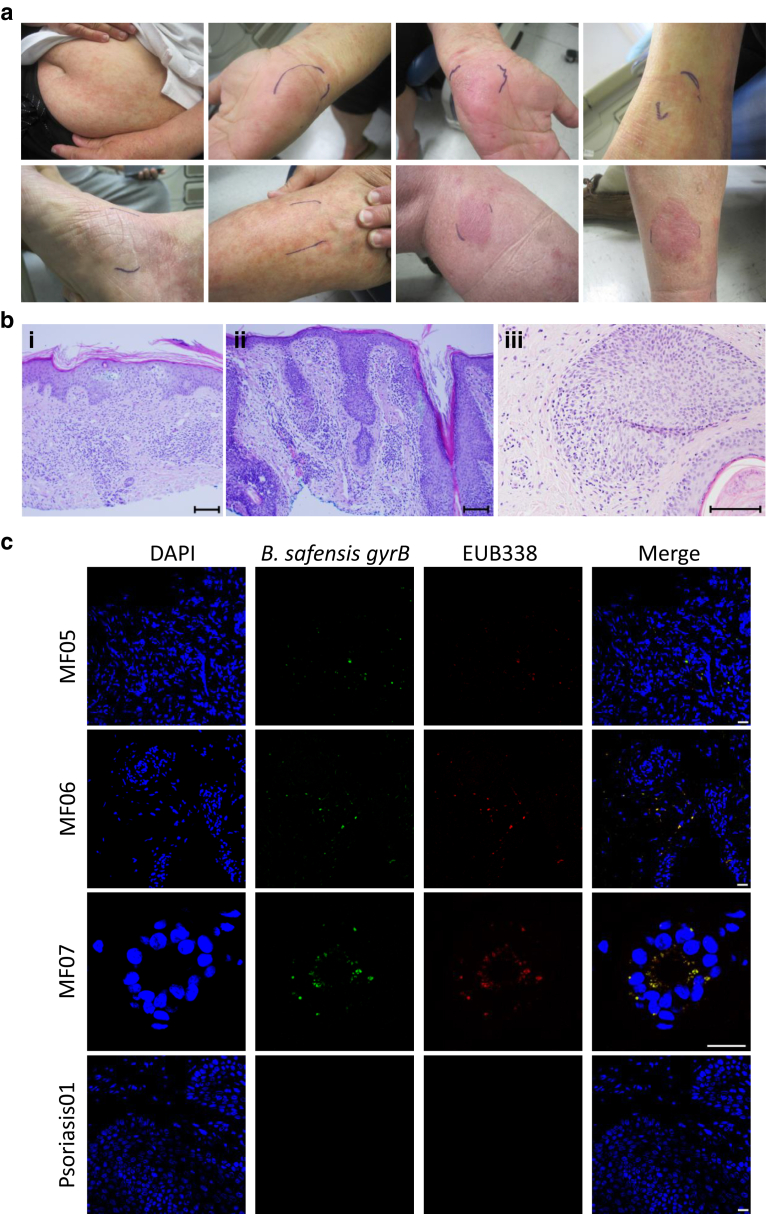
**Photographs, histology, and FISH of skin lesions from patients with CTCL sampled in this study.** (**a**) Representative lesional photos (upper and lower panel) from one representative patient (MF04). The region marked in blue represents the area that was swabbed. (**b**) Representative H&E sections of three patients at the time of diagnosis (MF05, MF06 and MF01). Bar = 100 μm. (**b**) The images show (i) the histology from a biopsy of the right lateral thigh with an atypical T cell infiltrate with exocytosis involving the epidermis, (ii) the histology from a biopsy of the right lateral periorbital region with features of folliculotropic mycosis fungoides (patient MF06), and (iii) the histology from a biopsy of the left buttock region with infiltration of hair follicles by atypical intrafollicular and perifollicular lymphocytes (together with additional clinical data supporting clonal T-cell receptor gene rearrangement; patient MF01). (**c**) FISH of skin biopsies from patients with MF and psoriasis. Cutaneous lesional biopsies from three patients with MF (MF05, MF06, and MF07) stain positive for a *gyrB-*specific 16S FISH probe (green) as well as for the eubacterial probe EUB338 (red). A merge of *gyrB* and eubacterial staining is shown in yellow. Cutaneous biopsies from psoriasis lesions were negative for *gyrB* (shown is one representative of two). Magnification for MF05, MF06, psoriasis = 40×; magnification for MF07 = 60×. Bar = 20 μm. CTCL, cutaneous T-cell lymphoma; MF, mycosis fungoides.

Next, we probed skin biopsies collected before microbiome swabs from our patient cohort for the presence of *B. safensis.* Skin biopsies were from lesional sites (Figure 9a and b) and the majority at the time of diagnosis (untreated stage). The 16S rRNA‒based FISH with a gyrB-specific gene probe (Branquinho et al., 2014a; Chen and Tsen, 2002; Wang et al., 2007, 2014b) was used to detect the presence of *B. safensis* (Figure 9c). Biopsies of skin from psoriasis (an inflammatory skin disease morphologically similar to early CTCL) served as controls. All of the three patient biopsies showed hybridization to the *gyrB* gene but to none of the psoriasis lesions, supporting a possible role for *B. safensis* early in the disease pathogenesis. Together with the culture- and sequencing-based studies, it can be concluded that the majority of patients with CTCL (86%, 6 out of 7 patients; see also Table 1) carry lesional *B. safensis* that was functionally linked to the stimulation of cutaneous and skin-homing blood T-cell proliferation and cytokine secretion, respectively (Figures 6, 7, and 8).

## Discussion

MF, the skin-limited type of CTCL, is thought to arise from malignant transformation of activated T cells in the setting of persistent antigenic stimulation and chronic inflammation (Burg et al., 2001; Girardi et al., 2004). Our data suggest increased *Bacillus* genus in patients with CTCL compared with that in healthy, body site‒matched skin. We identified the rare human skin commensal *B. safensis* only in patients with CTCL, predominantly within lesional skin. A recent skin microbiome survey of patients with CTCL from Finland, which was performed by whole-genome shotgun analysis, also supports the presence of *B. safensis* (base value: 6.95) and a phylogenetically close species, *B. pumilus* (base value: 19.3) (Salava et al., 2020). The phylogenetic relatedness of *B. pumilus* to *B. safensis* is shown in Figure 4. Furthermore, other related *Bacillus* species, *B. thuringiensis* and *B. licheniformis,* which we cultured from lesions of patients MF03 and MF06 in our study (Figure 2e)*,* were also present in another recent CTCL skin microbiome study from the United States (Harkins et al., 2021). Importantly, functional analyses of T-cell proliferation and cytokine secretion support a mechanistic role for *B. safensis* in the pathogenesis of MF. Its association with early CTCL and activation of CLA^+^ skin-homing T cells suggests that *B. safensis/pumilus* might serve as an initial instigator of CTCL that is likely exacerbated by more inflammatory events mediated by other microbiota at later stages. Mechanistically, one possible scenario is that *B. safensis* acts as an antigenic trigger, which initiates local expansion of *B. safensis‒*specific, skin-homing T cells and that this local expansion and cytokine release then increases inflammation together with other resident microbiota, resulting in Pautrier’s microabscesses. Because certain gut commensals thrive in the setting of inflammation (Chow et al., 2011), a feed-forward loop of inflammation and outgrowth of skin pathobionts seems plausible and needs to be tested in the future. Alternatively, T-cell secretion of IL-10 induced by *B. safensis* might be indicative of a regulatory T cell phenotype activated by STAT3 pathway in early stages of CTCL, thus allowing T cell expansion owing to immune evasion (Abraham et al., 2011; Krejsgaard et al., 2017). Further studies are needed to understand whether *B. safensis* acts directly on malignant T-cell clones or on bystander T cells in the tumor microenvironment.

It is notable that *B. safensis* has been shown to mediate transkingdom activity against *Candida albicans* by inhibiting its filamentation and biofilm formation through the degradation of *Candida* filaments (Mayer and Kronstad, 2017a, 2017b). This mechanism may support a preferential outgrowth of *B. safensis* within its niche. In addition, several studies showed that *B. safensis/pumilus* is also unusually resistant to UVR, which seems to be linked to certain putative DNA repair genes (Tirumalai et al., 2013). This could explain why some of our patients showed *B. safensis* colonization despite undergoing phototherapy and may also relate to relapses in some patients with CTCL.

**Figure 10:**
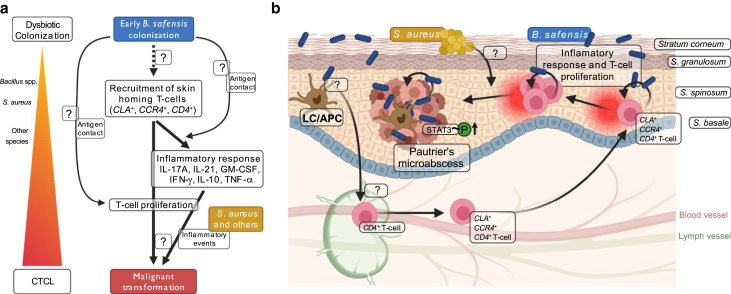
**Hypothesized involvement of *Bacillus safensis* and related strains in CTCL tumorigenesis.** (**a, b**) The hypothesized mechanistic role for *B. safensis* in the pathogenesis of MF—the most common form of CTCL—is schematically depicted in the left panel **a** and illustrated in the skin in the right panel **b**. *B. safensis* bacteria are indicated as blue rods, and *Staphylococcus aureus* bacteria are indicated as yellow circles. Cutaneous bacteria, *B.* *safensis* in this study, are taken up by professional antigen-presenting cells (APCs) such as Langerhans cells (LCs) that migrate to the local lymph node in order to present antigens to circulating T cells. Next, activated T cells are recruited to the skin via the skin homing markers CLA and CCR4. The direct antigen contact in the epidermis potentially triggers T cell proliferation and cytokine secretion activating STAT3 phosphorylation, thereby inducing a progressive inflammatory response that supports the malignant transformation of cutaneous T cells. Additional inflammatory events mediated by other microbiota (such as staphylococci, indicated by *S. aureus*) likely fuel this process, resulting in the formation of Pautrier’s microabscesses and ultimately promoting the progression of the T cell malignancy. Graphs were created with BioRender.com. APC, antigen-presenting cell; CLA, cutaneous lymphocyte‒associated antigen; CTCL, cutaneous T-cell lymphoma; LC, Langerhans cell; MF, mycosis fungoides; STAT3, signal transducer and activator of transcription 3.

Several investigations into *S. aureus* support that its toxins are responsible for fueling disease progression (Blümel et al., 2019; Willerslev-Olsen et al., 2016, 2013). This raises the question of whether *S. aureus* and *B. safensis* interact in skin lesions of patients with CTCL given that certain other *Bacillus* species can suppress *S. aureus* growth (Gonzalez et al., 2011). The presence of *B. safensis* could represent an antigenic trigger for the evolution from a polyclonal or oligoclonal adaptive immune response to a clonal malignant response in early CTCL lesions, with later *S. aureus* outgrowth acting as an innate immune driver of inflammation and CTCL pathogenesis through toxin production. More research on these scenarios is necessary, but *B. safensis* as a potential antigenic trigger can be inferred by our in vitro studies as summarized in Figure 10.

The antigens recognized by clonal T or B cells that arise in lymphomas remain largely unknown. A recent study identified lysolipids as targets recognized by plasma cells in myeloma (Nair et al., 2016), but the source of these antigens remains to be determined. Our findings support a paradigm, in which antigenic stimuli from a rare skin commensal may be a driver of lymphomagenesis in the skin of some patients with early CTCL akin to processes at mucosal sites in early mucosa-associated lymphoid tissue lymphoma. The limitations of our study include the limited number of subjects studied, the lack of exactly matched control subjects, and the isolation of all infiltrating cells from skin lesions without separating specifically malignant T cell clones, which is technically challenging. Larger cohorts and additional mechanistic research are needed to determine whether the recognition of *B. safensis* or closely related *Bacillus* species by CTCL T cells is a pathogenic feature in subsets of patients with CTCL. Furthermore, the specific antigen or antigens triggering malignant T cell activation in our study remain to be determined. Future studies utilizing in vivo and gnotobiotic models are necessary to test whether *B. safensis* is linked to CTCL pathogenesis in vivo. Similar to the therapeutic efficacy of *H. pylori* eradication in early mucosa-associated lymphoid tissue lymphoma, one can envision future therapeutic strategies aimed at skin commensals in this chronic debilitating skin condition with limited treatment options.

## Material and Methods

### Human subjects and microbiota sampling

All human subject protocols were approved by the Yale Human Investigations Committee and in accordance with the Declaration of Helsinki. Written informed consent was obtained from all study subjects. Exclusion criteria were ongoing chronic infection, antibiotic or probiotic use in the last 90 days, topical antibiotic or antimicrobial use in the last 7 days, bathing or tooth brushing in the last 8 hours, major gastrointestinal surgery in the last 5 years, gastrointestinal bleeding history, inflammatory bowel disease, bulimia or anorexia nervosa, morbid obesity, uncontrolled diabetes mellitus, malignancy in the past year (except CTCL), and known excessive alcohol use. Subjects with CTCL and healthy controls completed up to four study visits for the collection of detailed health and diet history; whole blood; and oral, lesional, and nonlesional skin microbiota sampling (ClinicalTrials.gov identifier: NCT02394964). Systemic lupus erythematosus and matched healthy donor samples served as additional controls. Lesional and adjacent nonlesional skin microbiota samples were collected as previously described (Greiling et al., 2018). The skin swab samples were rerun targeting the V1‒V3 16S rRNA region using the methods outlined in the section on 16S rRNA high-throughput sequencing. In brief, using sterile gloves, sterile Catch-All Swabs (EpiCentre Biotechnology, Madison, WI), that were premoistened in EpiCentre Yeast Cell Lysis Buffer, were rubbed vigorously on a 2‒3 cm^2^ area of skin for 60 seconds. Swabbed areas were marked (see Figure 9a). Nonlesional swabs were performed 2 inches away from the marked site and had to appear macroscopically uninvolved. In addition, air swabs taken before swabbing a skin site were analyzed to rule out environmental contaminations. Swabs were stored in Yeast Cell Lysis Buffer (EpiCentre Biotechnology) at ‒80 °C until DNA was extracted. For DNA extraction, swabs were incubated at 37 °C in EpiCentre Yeast Cell Lysis Buffer for 1 hour with shaking. Catch-all swabs were spun down in the same buffer, bead beaten using a BioSpec Mini-Beatbeater-16 with 0.1 mm glass beads (MP Biomedicals, Santa Ana, CA) for 2 minutes, and then incubated for 30 minutes at 65 °C. Samples were cooled on ice for 5 minutes, and then 250 μl of MPC Reagent (EpiCentre Biotechnology) was added to precipitate protein. Samples were centrifuged for 10 minutes, and an equal volume of 100% ethanol was added to the supernatant. Next, the standard protocol for the PureLink Genomic DNA mini kit (Invitrogen, Waltham, MA) was followed. DNA quantity and quality were determined by A_260_ and A_280_ on a NanoDrop 2000 spectrophotometer.

### 16S rRNA high-throughput sequencing

DNA isolation from microbiota samples was performed as described previously (Greiling et al., 2018; Ruff et al., 2019). The V1‒V3 regions of the 16S rRNA gene were PCR amplified, normalized, pooled, and sequenced using the Illumina MiSeq with 2 × 300 bp paired-end reads as described previously (Greiling et al., 2018). Analysis of 16S sequencing reads was performed as described before (Kozich et al., 2013; Ruff et al., 2019) with the following modifications: Quantitative Insights Into Microbial Ecology analysis was performed using version 2 (Bolyen et al., 2019) core distribution 2018.11.0, with denoising performed using the QIIME2 DADA2 (Callahan et al., 2016) plugin (trimLeft = 0 nucleotides for forward and reverse; truncLen = 260 nucleotides forward and 260 nucleotides reverse). Denoised and filtered ASVs were assigned taxonomy using a prefitted (Silva 132 [Quast et al., 2013] 99% OTUs full-length sequences) scikit-learn 0.19.1 (Pedregosa et al., 2011)‒based QIIME2 plugin. Sequences were further filtered to exclude unassigned and eukaryotic sequences. These filtered ASVs were rarefied to the lowest number of reads that included all samples for analysis while approaching the upper limit of diversity captured by Shannon H index, 3,030 sequences per sample. Demultiplexed and preprocessed V1‒V3 16S rRNA reference sequences were obtained from the National Institutes for Health human microbiome project QIIME SOP repository (Gevers et al., 2012). ASVs were assigned taxonomy, filtered, and rarefied to 3,030 sequences per sample as described earlier for the primary cohort.

### Nucleotide sequence alignment

Nucleotide sequences were aligned using Clustal Omega (Sievers et al., 2011). *B. safensis* FO-36b 16S rRNA NR_041794.1 served as known sequence control. Sequences were compared with those of a *B. safensis* culture isolate (patient MF05 with CTCL). ASVs were considered *B. safensis* hits if they had an exact or greater than 99% match to the cultured *B. safensis* strain*.*

### Phylogenetic analysis

A phylogenetic tree for the evolutionary history of *Bacillus* species was inferred using the Minimum Evolution method (Rzhetsky and Nei, 1992) with an optimal tree with the sum of branch length = 0.34457016. The evolutionary distances were computed using the Maximum Composite Likelihood method (Tamura et al., 2004) and are in the units of the number of base substitutions per site. The minimum-evolution tree was searched using the Close-Neighbor-Interchange algorithm (Nei and Kumar, 2000) at a search level of 1. The Neighbor-joining algorithm (Saitou and Nei, 1987) was used to generate the initial tree. The analysis involved 13 nucleotide sequences with the following GenBank identifiers: *B. altitudinis* SCU11 (CP038517.1:843690-845230), *B. anthracis* strain BF1 (CP047131.1:266707-268255), *B. cereus* ATCC 10987 (AE017194.1:279626-281133), *B. licheniformis* strain ATCC 14580 (CP034569.1:163688-165236), *B. pumilus* strain MTCC B6033 (CP007436.1:2780104-2781653), *B. safensis* FO-36b (CP010405.1:166221-167774), *B. subtilis* subsp. *subtilis* strain 168 (CP053102.1:96641-97941), *B. thuringiensis* strain ATCC 10792 (CP020754.1:4310655-4311955), *B. toyonensis* strain P18 (CP064875.1:82409-83709), *Cutibacterium acnes* strain ATCC 6919 (CP044255.1:597524-599046), *Pseudopropionibacterium propionicum* F0230a (CP002734.1:26370-27896), *S. aureus* strain ATCC BAA-39 (CP033505.1:542857-544410)*, S. capitis* strain FDAARGOS_378 (CP023966.1: 2171633-2173177), *S. cohnii* strain FDAARGOS_538 (CP033735.1: 1594593-1595894), *S. epidermidis* RP62A (CP000029.1:155527-157079), *S. hominis* strain 19A (CP031277.1: 792250-793795), *S. pasteuri* strain SP1 (CP004014.1: 440787-442334), *S. simulans* strain MR1 (CP015642.1: 888201-889502), and *S. warneri* strain 16A (CP031269.1: 752181-753483). All positions containing gaps and missing data were eliminated. There were a total of 1,258 positions in the final dataset. Evolutionary analyses were conducted in MEGA7 (Kumar et al., 2016).

### Bacterial culturing and Sanger sequencing

Lesional and adjacent nonlesional skin microbiota samples were collected using sterile gloves with sterile Catch-All Swabs and transported in individual tubes containing culture medium (Difco Nutrient Broth, catalog number 234000, BD, Franklin Lakes, NJ) or Trypticase-based medium (Culture Medium, BD). After 24 hours of growth, swabs were plated out on agar plates with the same medium; 16‒24 hours later, single colonies were picked and grown up for 8 hours, and DNA was extracted using the protocol for gram-positive bacteria (Dneasy, Qiagen, Hilden, Germany). Full-Length 16S rRNA PCR was run using the universal 16S rRNA primers 8 forward and 1,391 reverse and sequenced by Sanger sequencing. Cultures were grown to an optical density of 1; colony-forming units were calculated and frozen at ‒80 °C either in Nutrient Broth (Difco Nutrient Broth; catalog number 234000, BD) or Trypticase-based medium (Culture Medium, BD) for in vitro stimulations. As controls for in vitro proliferation assays, *D. grandis* and *A. radioresistens* were purchased from the German Collection of Microorganisms and Cell Cultures (Braunschweig, Germany) (DSMZ # 3963 and #6976, respectively).

### T cell isolation from skin biopsies

Selected lesional biopsies were taken prospectively from patients with CTCL if visible skin lesions were present as determined by certified medical personnel using sterile, single-use materials and transported on ice, followed by T cell extraction per published protocols (Clark et al., 2006b). In brief, cell foam matrices (grids, Cytomatrix Pty, Hawthorn East, Victoria, Australia) were coated with collagen (250 μl collagen [BD Biosciences, Franklin Lakes, NJ]/10 ml PBS). Skin fragments were moved to skin T-cell medium (400 ml Iscove’s medium [Thermo Fisher Scientific, Schwerte, Germany], 100 ml FBS, 5 ml L-Glutamine [Invitrogen], 5 ml penicillin/streptomycin, 1.75 μl 2-mercaptoethanol), any hair was shaved off, and then skin fragments were minced into very small fragments. A 24-well plate with 2 ml of skin T-cell medium received fungizone (maximum of 1 week). The skin fragments were pressed onto the coated grids and put into an incubator (37 °C, 5% carbon dioxide). Medium changes occurred three times a week by gently aspirating the consumed medium. IL-2 (100 U/ml) and IL-15 (20 ng/ml) were added to stimulate expansion. Cells were carefully harvested using a pipette and were washed in 50 ml Hank’s/4-(2-hydroxyethyl)-1-piperazineethanesulfonic acid. Cells were kept in a nonsupplemented medium (without cytokines) before stimulation for at least 24 hours.

### Western blot

A total of 15 μg of lysed minced skin fragments were subjected to gel electrophoresis and blotted according to the manufacturer’s instructions after measurement of protein amount using the bicinchoninic acid method (Thermo Fisher Scientific). Phosphorylated STAT3 antibody (D3A7, Cell Signaling Technology, Danvers, MA) or total STAT3 antibody (clone D3Z2G, Cell Signaling Technology) were incubated overnight as primary antibodies in 5% BSA in PBS Tween-20 at a dilution of 1:1,000. Anti-human IgG horseradish peroxidase as a secondary antibody was used at a dilution of 1:1,000 in PBS, and blots were developed using enhanced chemiluminescence. Band intensities were measured using ImageJ (National Institutes of Health, Bethesda, MD).

### T cell proliferation and cytokine immunoassay

PBMCs were isolated from whole blood by Lymphoprep (Stemcell Technologies, Vancouver, Canada) gradient centrifugation. PBMCs were immunomagnetically separated using the following kits (Stemcell Technologies) per manufacturers’ instructions: monocytes using the EasySep Human CD14 Positive Selection kit and CD4^+^ T cells using the EasySep Human CD4^+^ T Cell Isolation kit. Selected cells were cooled in 90% heat-inactivated human AB serum with 10% dimethyl sulfoxide to ‒80 °C at ‒1 °C/minute and transferred to liquid nitrogen within 24 hours. Autologous monocytes were used as antigen-presenting cells for the T-cell library assay. Viable CLA^+^, CCR4^+^ memory (CD45RA^−^CD45RO^+^CD25^−^CCR4^+^CLA^+^) CD4^+^ T cells (antibodies from BioLegend, San Diego, CA) were sorted on a FACSAria machine (BD Biosciences). CD25-targeted depletion was performed using FACS sorting to exclude the CD25^+^ regulatory T cells in this study.

Cutaneous T cells or CD4^+^ T cells from peripheral blood were used for in vitro stimulation using the following protocol: 10,000 T cells were stimulated in vitro using monocytes that were pulsed for 3 hours with cultured heat-killed bacteria (10 bacterial cells/one monocyte at 65 °C for 15 minutes). Negative control wells contained monocytes only to assess any background signal. After 72 hours, culture supernatants were removed for cytokine measurements using a bead-based immunoassay (Luminex by MilliporeSigma, Burlington, MA) following the manufacturer`s instructions and as previously published (Greiling et al., 2018; Ruff et al., 2019). Cell proliferation was measured by nonradioactive ATP measurement using the ATP lite kit (PerkinElmer, Waltham, MA) following the manufacturer`s instructions and as previously published (Greiling et al., 2018; Ruff et al., 2019).

### 16s rRNA FISH on human skin tissue

The 16S rRNA‒targeted oligonucleotide probes used in this study were generated by biomers.net following a previously published *gyrB*-specific probe (Branquinho et al., 2014a; Chen and Tsen, 2002) and a previously published eubacterial probe EUB338 (Amann et al., 1990). The *gyrB* gene has been associated with certain strains other than *B. safensis*. However, besides the phylogenetically highly related *B. pumilus* (100% 16S rRNA identity; see also Fig. 4), only *B. cereus* was previously reported in human skin (Henrickson, 1989). For in situ hybridization, the probes were labeled with either FITC or Cy3 by biomers.net. Formalin-fixed, paraffin-embedded skin biopsies were obtained from the Dermatopathology laboratory at Yale University (New Haven, CT) and were deparaffinized in xylene and absolute ethanol for FISH studies. We used skin biopsies of two patients with psoriasis from a previous study (Greiling et al., 2018) as controls (none of them was on phototherapy).

Hybridizations were performed at 46 °C for 2 hours with hybridization buffer (0.9 M sodium chloride, 20 mM Tris-hydrogen chloride [pH 7.5], 0.05% sodium dodecyl sulfate, 20% formamide) containing 0.5 ng/μl of each labeled probe. A washing step was done at 46 °C for 10 minutes with washing buffer (0.215 M sodium chloride, 20 mM Tris-hydrogen chloride [pH 7.5], 0.05% sodium dodecyl sulfate, 0.025 mM EDTA). Slides were air dried and then mounted using Antifade Mounting Media with DAPI. Finally, the slides were visualized with a Leica Confocal Microscopy.

### Statistical analysis

Plotting of data and statistical analysis were performed using GraphPad Prism (GraphPad Software, La Jolla, CA). Unless otherwise stated, statistical significance was determined by unpaired two-tailed Student’s *t*-test, and differences were considered statistically significant if *P* < 0.05. *P*-values are represented using ∗ for *P* < 0.05, ∗∗ for *P* < 0.01, ∗∗∗ for *P* < 0.001, and ∗∗∗∗ for *P* < 0.0001.

### Data availability statement

Datasets related to this article have been deposited to the European Nucleotide Archive and can be found under the accession number ERP125433 or through the following link: https://www.ebi.ac.uk/ena/browser/view/PRJEB41619?show=reads).

## ORCIDs

Carina A. Dehner: http://orcid.org/0000-0001-5214-4813

William E. Ruff: http://orcid.org/0000-0001-5828-6425

Teri Greiling: http://orcid.org/0000-0002-0028-8986

Márcia S. Pereira: http://orcid.org/0000-0002-8708-8388

Sylvio Redanz: http://orcid.org/0000-0003-1541-2545

Jennifer McNiff: http://orcid.org/0000-0001-8142-6481

Michael Girardi: http://orcid.org/0000-0003-1887-9343

Martin A. Kriegel: http://orcid.org/0000-0002-7371-0391

## Author Contributions

Conceptualization: CAD, WER, MG, MAK; Data Curation: CAD, WER; Formal Analysis: CAD, WER, JM, SR; Funding Acquisition: MAK; Investigation: CAD, WER, TG; Methodology: CAD, WER, TG, MG; Project Administration: MAK, MG; Resources: JM, TG; Supervision: MAK; Validation: CAD, WER; Visualization: CAD, SR; Writing - Original Draft Preparation: CAD, WER, SR, MG, MAK; Writing - Review and Editing: CAD, WER, MSP, MG, MAK
